# Supplementary material for: WordCommentsAnalyzer: A windows software tool for qualitative research
Source: F1000Res. 2018 Sep 4;7:536. Originally published 2018 May 3. [Version 2] doi: 10.12688/f1000research.14819.2 (PMC6137411; doi:10.12688/f1000research.14819.2)
Supplement: Supplementary file 3 [file f1000research-7-17614-s0002.tgz › b2a79496-4ada-47c4-9682-a99ce867836d.zip!/Eight Iranian Health Journals/Journal of Knowledge and Health_35.docx]

© Journal of Knowledge and Health. This work is licensed under CC BY 4.0 (https://creativecommons.org/licenses/by/4.0/).

Volume & Issue: دوره 4، شماره 3: 1388

Title: برآورد بقای نسبی بیماران مبتلا به سرطان پستان مراجعه¬کننده به انستیتو كانسر بیمارستان امام خمینی طي سال¬های 74-1369

http://knh.shmu.ac.ir/index.php/site/article/view/178

مقدمه: نسبت بقای نسبی به­عنوان برآوردی از بقای خالص معیار مناسب­تری در مقایسه با بقای مشاهده شده و بقای علت ویژه برای تحلیل بقای بیماران سرطانی بر پایه اطلاعات مراکز ثبت سرطان مبتنی بر جمعیت می­باشد. این پژوهش با هدف برآورد و بررسی وضعیت بقای خالص بیماران مبتلا به سرطان پستان انجام شده است. مواد و روش­ها: برای محاسبه نسبت بقای نسبی در این کوهورت تاریخی، بقای مورد انتظار بر اساس اطلاعات سالانه مرگ در کشور فنلاند، به­عنوان جمعیت استاندارد، برآورد شده است و تعداد 662 بیمار زن با تشخیص سرطان پستان، بر اساس گزارش­های پاتولوژی، که در دوره تقویمی 74-1369 به انستیتو كانسر بیمارستان امام خمینی مراجعه و تا پایان سال 1379 پیگیری شده­اند مورد تحلیل قرار گرفته­اند. در این پژوهش برای محاسبه نسبت بقای نسبی از روش دوم ادیرر استفاده شده است. داده­های پژوهش با برنامه­نویسی تحت نرم­افزار SAS مورد تجزیه و تحلیل قرار گرفت. نتایج: نتایج حاصله نشان می­دهد نسبت بقای نسبی سه ساله برای گروه­های سنی 44- 15، 59- 45 و 74- 60 به­ترتیب برابر 85%، 90% و 80% و برای گروه سنی بالاتر از 75 سال برابر 67% بسیار متفاوت از دیگر گروه­هاست. برآورد نسبت بقای نسبی برای گروه سنی 59- 45 برای دو سال متوالی تقریباً برابر با یک به­دست آمد. برای برخی فواصل پیگیری برآورد بقای نسبی بیش از یک به­دست آمد.نتیجه­گیری: بر اساس نتایج، احتمال بقا با افزایش گروه­های سنی کاهش می­یابد. این کاهش برای گروه سنی بالاتر از 75 سال قابل ملاحظه است. هم­چنین در گروه­های سنی با افزایش سال­های پیگیری احتمال بقا ابتدا کاهش و سپس اندکی افزایش می­یابد. برای گروه سنی 45 تا 59 نقطه درمان آماری قابل پذیرش است و بقای نسبی در برخی فواصل برای گروه سنی 45 تا 59 و 60 تا 74 بیانگر کیفیت پیگیری نامناسب می­باشد

Title: اثر برنامه منتخب پیاده¬روی بر چگالی استخوانی، ترکیب بدن و استروژن سرم دختران چاق

http://knh.shmu.ac.ir/index.php/site/article/view/207

مقدمه: پوکی استخوان و چاقی، دو اختلال مهم ترکیب بدن، در حال شیوع می­باشند و تحقیقات کمی ارتباط بین چاقی و چگالی استخوانی با فعالیت بدنی را در دختران چاق مورد بررسی قرار داده­اند، بنابراین هدف از این پژوهش بررسی اثر برنامه پیاده­روی در پیشگیری از پوکی استخوان و کاهش چاقی در دختران چاق است. مواد و روش­ها: 20 نفر از دختران چاق غیر ورزشکار به­طور داوطلبانه در این پژوهش شرکت کردند و سپس به­طور تصادفی به دو گروه 10 نفره شاهد و مداخله تقسیم شدند در (گروه شاهد kg/m2 6/3±9/30=BMI و در گروه مداخله: kg/m2 8/1±2/30=BMI). ابتدا و بعد از 2 ماه، چگالی استخوانی، ترکیب بدن و استروژن سرم آزمودنی­ها اندازه­گیری شدند. سپس گروه مداخله برنامه تمرینی را که شامل 30 دقیقه پیاده­روی با شدت 75%- 50% ضربان قلب، 3 جلسه در هفته و به مدت2 ماه بود، انجام دادند. اطلاعات به­دست آمده با آزمون آماری t مستقل و در سطح 05/0P< تجزیه و تحلیل شدند. نتایج: نتایج این پژوهش نشان دادکه چگالی استخوانی در دو ناحیه ران و ستون فقرات در دو گروه مداخله و شاهد بعد از 2 ماه تغییر معناداری نداشتند (05/0<(P، اما تمام اجزای ترکیب بدن (001/0P=) و سطح استروژن سرم (035/0P=) نسبت به گروه شاهد به­طور معناداری تغییر یافتند. نتیجه­گیری: به­عنوان یک نتیجه احتمالی از تحقیق حاضر می­توان چنین پیشنهاد کرد که پیاده­روی طولانی مدت و با شدت بیش­تر سبب تأثیر مثبت بر چگالی استخوان و عوامل مؤثر بر آن در دختران چاق می­شود ولی به هر حال این نتیجه­گیری به تحقیقات بیش­تر نیاز دارد.

Title: بررسی ارتباط نشانگرهای اقتصادی- اجتماعی با وضعیت تغذیه دانشجویان ساکن خوابگاه¬های دانشگاه علوم پزشکی شیراز

http://knh.shmu.ac.ir/index.php/site/article/view/210

مقدمه: با توجه به این­که تغذیه یکی از عوامل مهم و مؤثر بر سلامت و تندرستی است و این مسأله در مورد دانشجویان حائز اهمیت بسیار زیادی است، این مطالعه عوامل اقتصادی- اجتماعی مؤثر بر وضعیت تغذیه دانشجویان ساکن خوابگاه­های دانشگاه علوم پزشکی شیراز را بررسي مي­نمايد. مواد و روش­ها: در این مطالعه مقطعی به روش نمونه­گیری چند مرحله­اي تصادفی 405 دانشجو، انتخاب و با استفاده از پرسش­نامه بررسی وضعیت تغذیه مورد ارزیابی قرار گرفتند. تجزیه و تحلیل اطلاعات با استفاده از آزمون آنالیز واریانس یک­طرفه و نمودار لورنز انجام شده است. برای انجام آنالیزهای مربوطه از نرم­افزار stata استفاده شد. نتایج: مقادير دريافتي كربوهيدرات، پروتئين، ويتامين B6، B12 و فسفر با (05/0>P) و مقادير دريافتي ويتامين C، ويتامين A و آهن با (001/0>P) با جنسيت رابطه معناداری داشتند. مقادير دريافتي ويتامين C و ويتامين A، فسفر و آهن با مقطع تحصيلي ارتباط معناداری داشتند. همبستگي مثبت بين تعداد سال­هاي سكونت در خوابگاه با مقادیر دریافتی ويتامين C با (2/0=r و 001/0>P) و كلسيم با (1/0=r و 05/0>P) وجود داشت. ارتباط درآمد خانوادگی با شاخص ترکیبی وضعیت دریافت ریزمغذی­ها و درشت­مغدی­ها معنادار بود (014/0±054/0). نتیجه­گیری: در این مطالعه تحصیلات والدین و درآمد خانواده از عوامل مؤثر بر الگوی تغذیه دانشجویان بودند. هم­چنین جنس به­عنوان یک عامل تعیین­کننده­ای در دریافت بعضی ریزمغذی­ها و درشت مغذی­ها در دانشجویان ساکن خوابگاه عمل می­کرد.

Title: بررسی اپیدمیولوژیک سل ریوی طی سال¬های 1383 تا 1386 در شهرستان ارو

http://knh.shmu.ac.ir/index.php/site/article/view/216

مقدمه: بيماري سل يكي از قديمي­ترين و شناخته شده­‌ترين بيماري­ها است كه از ساليان دور با بشر همراه بوده است. میزان بروز سل در طی دهه­های قبل مجدداً افزایش یافته است. هدف این مطالعه، بررسی اپیدمیولوژیک سل ریوی طی سال­های 1383 تا 1386 در شهرستان ارومیه می­باشد. مواد و روش­ها: این مطالعه به­صورت توصیفی- تحلیلی می­باشد. که از اطلاعات پرونده­های بیماران مبتلا به سل ریوی تشخیص داده شده طی سال­های 1383 تا 1386 استفاده شده است. در این مطالعه از نرم­افزار SPSS و WinPepi جهت آنالیز داده­های جمع­آوری شده استفاده شده است. نتایج: بر طبق یافته­های این مطالعه در طی سال­های 1383 تا 1386، تعداد 248 بیمار مبتلا به سل ریوی در شهرستان ارومیه شناسایی شدند که از این تعداد 151 مورد اسمیر مثبت و 97 مورد اسمیر منفی می­باشند (میزان بروز چهار ساله = 54/29 به ازای صد هزار نفر). از نظر توزیع جنسی 164 نفر (1/66%) مذکر و 84 نفر (9/33%) مؤنث بودند. میزان بروز از 55/6 در صد هزار نفر در سال 83 به 2/8 در صد هزار نفر در سال 86 افزایش یافته است ولي اين روند افزايشي از نظر آماري معنادار نمي­باشد (22/0 =Trend P.V). نتیجه­گیری: نتایج این مطالعه نشان می­دهد که میزان بروز سل ریوی اسمیر مثبت بیش­تر از سل ریوی اسمیر منفی (تقریباً یک­ونیم برابر) می­باشد و میزان بروز سل ریوی طی چهار سال مورد مطالعه افزایش یافته است.

Title: بررسی آگاهی، نگرش و عملکرد تغذیه¬ای دانش¬آموزان دختر مقطع راهنمایی ناحیه 4 شهر اصفهان در سال 1387

http://knh.shmu.ac.ir/index.php/site/article/view/225

مقدمه: هدف از این بررسی تعیین آگاهی، نگرش و عملکرد تغذيه­اي دانش­آموزان دختر مقطع راهنمایی در ناحیه 4 اصفهان است. مواد و روش­ها: این مطالعه به­روش توصیفی و بر روی 120 نفر از دانش­آموزان انجام شد. به­منظور ارزیابی آگاهی، نگرش و عملکرد تغذیه­ای افراد از پرسش­نامه 3 قسمتی (مشخصات دموگرافیک- آگاهی- مدل بزنف) استفاده شد که 3 قسمت اول آن به­وسیله خود گزارش­دهی و قسمت عملکرد به­وسیله مصاحبه تکمیل گردید. قسمت عملکرد نیز شامل یاد آمد 24 ساعته خوراک بود که در 3 روز و به وسیله مصاحبه تکمیل گردید. نتایج: نتایج نشان می­دهد که 5/37% دانش­آموزان در مورد تغذیه سالم، آگاهی ضعیف داشتند در حالی­که 90% از دانش­آموزان عملکرد تغذیه­ای نامطلوب داشتند. نتایج حاصل از قسمت­های مختلف مدل بزنف نیز نشان داد که میانگین نگرش نسبت به ارزیابی رفتار 6/74، نگرش نسبت به رفتار 6/64، اعتقادات نرمی 8/72، نرم­های انتزاعی 9/58، عوامل قادرکننده 2/54 و قصد رفتار 9/68 بوده است. نتیجه­گیری: با توجه به یافته­های این مطالعه مشخص است که درصد کمی از نوجوانان از عملکرد تغذیه مطلوب برخوردارند و در بسیاری از موارد نیز عملکرد آن­ها مطابق دانش تغذیه­ای نیست؛ این یافته­ها ضرورت مداخلات مداوم را بر اساس مدل بزنف در دانش­آموزان نشان می­دهد.

Title: بررسی کفایت برنامه درسی بالینی رشته مامایی در دستیابی دانشجویان به اهداف یادگیری پایه از دیدگاه دانشجویان و اساتید

http://knh.shmu.ac.ir/index.php/site/article/view/227

مقدمه: دستيابي به استانداردهاي حرفه­اي در علوم پزشكي و از جمله در حرفه مامايي از طريق حصول اهداف مهارت باليني حرفه صورت مي­پذيرد. با توجه به اهمیت برنامه درسی بالینی، مطالعه حاضر با هدف تعیین نظرات دانشجویان و اساتید رشته مامایی در خصوص کفایت برنامه درسی بالینی در دستیابی دانشجویان به اهداف یادگیری پایه طرح­ریزی و اجرا شده است. مواد و روش­ها: این پژوهش یک مطالعه توصیفی- تحلیلی است كه در دانشگاه علوم پزشكي شاهرود و در سال 1387 انجام گرديد. نمونه­هاي پژوهش شامل 57 نفر از دانشجویان سال آخر و 9 نفر از اساتید این رشته بودند. پرسش­نامه پژوهش سه درس اختصاصی "بارداری"، زایمان" و "زنان" را با 39 گویه مورد سنجش قرار می­داد. پاسخ­های افراد شرکت­کننده در مقیاس لیکرت از کاملاً تا اصلاً متفاوت بود. نتایج: بررسي و مقايسه دو گروه واحدهاي شركت­كننده در پژوهش نشان داد كه آن­ها ميزان دستيابي به اهداف آمورشي باليني را به شكل متفاوتي ارزيابي نمودند. میانگین نمرات به خود در بین دانشجویان در سه درس فوق به­ترتیب 2/18±0/65، 8/24±9/50 و 5/25±4/54 با نمره کل 6/20±7/58 بود. نتایج وجود اختلاف معنادار را بین نمرات دانشجویان و اساتید در سه درس مذکور نشان می­دهد. نتیجه­گیری: با توجه به نتایج، به نظر می­رسد که ایجاد تغییرات وسيع در محیط­های بالینی و آموزشی نظیر جلب همکاری بیش­تر پرسنل باليني با سيستم آموزشي و دانشجویان، جلب پذيرش بیماران نسبت به مشارکت بالینی فعال­تر دانشجویان در امر درمان ضروري باشد تا به احساس كفايت بيش­تري در دانشجويان بيانجامد.

Title: کاهش عوامل خطر انعقادی، اکسیداتیو، آپولیپوپروتئین و پیشرفت آترواسکلروز تحت تأثیر مصرف سرکه سیب در خرگوش¬های هایپرکلسترولمیک

http://knh.shmu.ac.ir/index.php/site/article/view/230

مقدمه: سرکه سیب یک ترکیب آنتی­اکسیدان است و دارای مصارف دارویی زیادی می­باشد. در این مطالعه اثرات سرکه سیب بر تعدادی از عوامل خطر بیوشیمیایی و پیشرفت آترواسکلروز در خرگوش­های هایپرکلسترولمیک بررسی شده است. مواد و روش­ها: 32 خرگوش نر نیوزیلندی به­طور تصادفی به 4 گروه تقسیم­بندی شدند: گروه رژیم معمولی، گروه رژیم پرکلسترول (1% کلسترول)، گروه رژیم پرکلسترول (1% کلسترول) و ml 5 سرکه سیب، گروه رژیم پرکلسترول (1% کلسترول) و ml 10 سرکه سیب. فاکتورهای MDA (مالون دی آلدئید)، oxLDL (LDL اکسید شده)، فیبرینوژن، فاکتور هفت (VII) و آپولیپوپروتئین­های A و (ApoA, ApoB) B، قبل از مداخله و در انتهای دوره (2 ماه) اندازه­گیری شدند. در انتهای مطالعه، تشکیل رگه چربی در آئورت با استفاده از متد  چکانوف در همه گروه­ها تعیین و بررسی شد. نتایج: مصرف هر دو دوز سرکه سیب موجب کاهش معنادار در سطح فاکتورهای فیبرینوژن، oxLDL، MDA، ApoB/ApoA، ApoB نسبت به گروه رژیم پرکلسترول شدند (05/0>P) ولی تأثیری بر ApoA نداشتند (05/0<P). هم­چنین مصرف سرکه سیب به ميزان زيادي موجب كاهش ضايعه در ديواره آئورت نسبت به گروه پركلسترول شد. نتیجه­گیری: نتایج این مطالعه پیشنهاد کننده تأثیر حمایتی مصرف سرکه سیب (به­عنوان یک آنتی اکسیدان) بر روی برخی از عوامل خطر آترواسکلروز می­باشد.

Title: بررسی اپیدمیولوژیک موارد حیوان گزیدگی در شهرستان شاهرود

http://knh.shmu.ac.ir/index.php/site/article/view/232

مقدمه: هاری، بیماری عفونی دستگاه عصبی مرکزی است که همه پستانداران و انسان را مبتلا می­کند. مطالعه حاضر به­منظور بررسی اپیدمیولوژی بیماری هاري در شهرستان شاهرود صورت گرفته است. مواد و روش­ها: در اين مطالعه توصیفی داده­های مرتبط با کلیه موارد حیوان گزیدگی در شهرستان شاهرود در سال 1387 بر اساس اطلاعات موجود در دفاتر ثبت موارد حیوان­گزیدگی شهرستان جمع­آوری گردید. نتايج: در طي سال 1387 كل موارد حيوان گزيدگي 588 مورد بوده كه اکثریت آن­ها (1/82%) مرد بوده و محل سكونت 7/35%  افراد شهر و 3/64% روستا بود. تنها دو مورد از موارد حيوان­گزيده غيرايراني بودند. میزان بروز در شهر 129 و در روستا 497 در صد هزار نفر گزارش شده که به­طور معناداری بیش­تر از شهر می­باشد. از كل موارد مورد گزش، 159 نفر (27%) در شهر و 429 نفر (73%) در روستا مورد گزش حيوانات قرار گرفته بودند. نوع حيوانات گزنده در اغلب موارد سگ (1/79%) و گربه (6/12%) بوده و گرگ، روباه و شغال در 12 نفر از موارد به­عنوان عامل گزش گزارش شده­اند. همه موارد گزش، واكسيناسيون خود را كامل نموده و در 3/82% حيوان گزنده پس از 10 روز زنده و 2% مرده و 6/15% وضعيت حيوان گزنده پس از 10 روز نامعلوم گزارش شده است. ميزان بروز ساليانه حيوان گزيدگي در شهرستان، 246 در صدهزار نفر محاسبه گردید. نتيجه­گيري: حيوان­گزيدگي هنوز يكي از مشكلات مهم بهداشت عمومي است كه فعاليت­هاي آموزشي همراه با بهبود همكاري­هاي برون­بخشي مي­تواند نقش به­سزايي در كنترل موارد آن داشته باشد.
